# Novel Trends into the Development of Natural Hydroxyapatite-Based Polymeric Composites for Bone Tissue Engineering

**DOI:** 10.3390/polym14050899

**Published:** 2022-02-24

**Authors:** Diana-Elena Radulescu, Ionela Andreea Neacsu, Alexandru-Mihai Grumezescu, Ecaterina Andronescu

**Affiliations:** 1 Department of Science and Engineering of Oxide Materials and Nanomaterials, Faculty of Applied Chemistry and Materials Science, University Politehnica of Bucharest, 011061 Bucharest, Romania; radulescu.diana95@gmail.com (D.-E.R.); grumezescu@yahoo.com (A.-M.G.); ecaterina.andronescu@upb.ro (E.A.); 2 Academy of Romanian Scientists, 54 Independentei, 050094 Bucharest, Romania; 3 National Research Center for Micro and Nanomaterials, Faculty of Applied Chemistry and Materials Science, University Politehnica of Bucharest, 060042 Bucharest, Romania; 4 Research Institute of the University of Bucharest (ICUB), University of Bucharest, 050657 Bucharest, Romania

**Keywords:** bone tissue engineering, hydroxyapatite, polymer, scaffold, bioactivity

## Abstract

In recent years, the number of people needing bone replacements for the treatment of defects caused by chronic diseases or accidents has continuously increased. To solve these problems, tissue engineering has gained significant attention in the biomedical field, by focusing on the development of suitable materials that improve osseointegration and biologic activity. In this direction, the development of an ideal material that provides good osseointegration, increased antimicrobial activity and preserves good mechanical properties has been the main challenge. Currently, bone tissue engineering focuses on the development of materials with tailorable properties, by combining polymers and ceramics to meet the necessary complex requirements. This study presents the main polymers applied in tissue engineering, considering their advantages and drawbacks. Considering the potential disadvantages of polymers, improving the applicability of the material and the combination with a ceramic material is the optimum pathway to increase the mechanical stability and mineralization process. Thus, ceramic materials obtained from natural sources (e.g., hydroxyapatite) are preferred to improve bioactivity, due to their similarity to the native hydroxyapatite found in the composition of human bone.

## 1. Introduction

Bone tissue engineering (BTE) has gained great interest in the last decade, as the frequency of degenerative diseases or tissue-damaging has increased [1]. Hence, the applications of BTE focus on the restoration of the native bone’s functionality by augmenting its regeneration rate with the aid of cells, osteogenic factors, or biomaterials. The perfect solution in improving bone recovery is represented by developing a biomaterial with good biological activity and mechanical properties that can maintain tissue functions [2,3].

**Figure 1:**
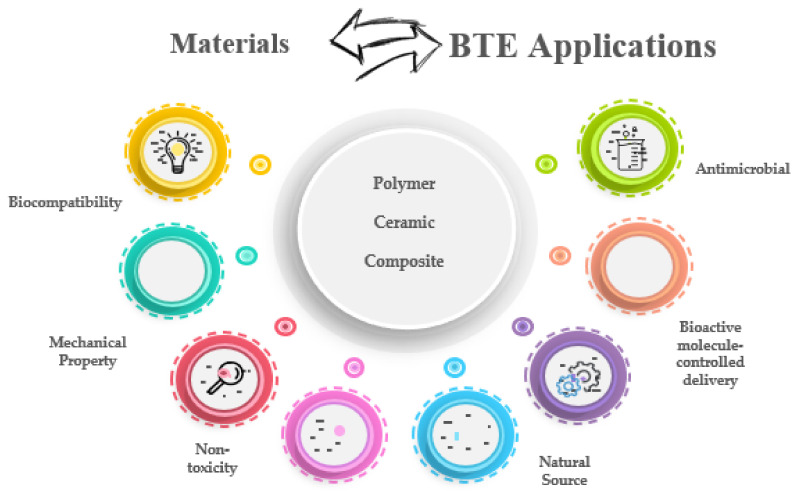
Schematic representation of the relationship between materials and necessary properties for BTE scaffolds.

Scaffolds are three-dimensional (3D) structures that need to meet specific criteria for BTE: biocompatibility, high surface area, appropriate mechanical properties for bone, enhance cell adhesion and lead to new tissue formation [3,4]. For the moment, the use of a single material as a scaffold is difficult to be further applied, as it cannot fully meet the requirements for BTE applications, as shown in (Figure 1). Therefore, the latest studies are currently concentrated on the development of composite materials, based on polymers and ceramics, that can mimic the composition of osseous tissue [5]. In this regard, biopolymers are known for their superior biocompatibility and good processability. Further, the mechanical strength is reduced, and the degradation rate is according to the osteogenesis rate, leading to pH modifications of the adjacent environment, generated by degradation products. On the other hand, even though bioceramics have good bone conductivity and biocompatibility, there is still a problem regarding the low toughness and significant brittleness that leads to damage and poor use reliability [5,6]. The limitations mentioned above have been addressed by developing biomaterials characterized by biomimicry and tissue regeneration capacity. The bioresorbability and bioactivity can be merged into composite materials to overcome the drawbacks of bioceramics and biopolymers. The obtained scaffold should be able to meet the complex requirements of tissue engineering, progressing from bone substituting materials to bone regeneration. These bioactive materials must have good biomechanical stability, biocompatibility, non-toxicity, and good processability to stimulate natural bone regeneration, by encapsulating inductive factors that promote bone healing [7,8,9].

Osseous tissue is a complex composite that can be reinforced with the aid of metallic, ceramic, composite, or polymeric materials. In this regard, bioceramic materials are the most promising materials for implantation due to their resemblance with bone composition. Moreover, calcium phosphates (CaPs) have been widely used as implantable materials due to their outstanding biocompatibility and similar chemical properties with hard tissues [10,11]. Additionally, CaP materials are essential for hard tissue reconstruction applications due to their outstanding biocompatibility, bioactivity, non-immunogenicity, non-toxicity, and non-inflammatory behavior [12]. Until this moment, numerous types of hydroxyapatite-based materials (HA-based materials), such as pure HA, HA/polymer composites, and ion-doped HA, have been investigated and designed, although some disadvantages still exist. Therefore, there is still a long way to go before obtaining satisfying HA-based materials [13,14,15].

Nowadays, already chemically synthesized HA has been used for BTE, but its poor durability and stability have reduced its feasibility into biomedical applications. These drawbacks of physically and chemically synthesized HA led to the use of natural biowastes to synthesize HA. Recently, a unique and efficient strategy for preparing functionalized biomaterials with complex structures obtained from natural origins has been demonstrated. Thus, organic food waste, such as bovine/fish bones, seashells, and eggshells, could have perfect potential in obtaining HA, with very high availability that could enhance orthopedic applications [16,17]. Further, to improve the bioactivity of the developed materials, scaffolds can be obtained by combining biodegradable polymers (for adaptive degradation and biocompatibility) and bioceramics (for strength and bioactivity) successfully [18,19].

This study aims to present the most used polymers in BTE, that can be further combined with ceramic materials. The main highlight of this research is represented by the use of HA obtained from natural sources to increase the bioactivity of composite scaffolds. Further, the required properties of composites for the proper functionality of scaffolds, and recent applications, will be briefly described to select the ideal material for orthopedic applications.

## 2. Polymers Applied in BTE

Over the years, it was concluded that the properties of biomaterials must be thoroughly investigated before considering them for BTE applications. These include chemical composition, structural and biological characteristics, degradation rate, and processability. Thus, polymeric scaffolds presented great interest due to their distinguishing features, such as the capacity to provide osteoinductive support to the transplanted or native cells at the injured zone, harbor multiple spatio-temporal cues, degradation rate similar to osteogenesis rate, and capacity to integrate with native tissue throughout wide chemical modifications. Considering their source of provenience, polymers applied in BTE can be classified as synthetic or natural polymers [20].

Several natural biopolymers, such as collagen (Col), chitosan (CS), silk fibroin (SF), cellulose, alginate, and hyaluronic acid, have been intensively researched, considering their present incorporation into other nanomaterials due to their increased bioactivity. On the other hand, synthetic polymers have also been presented in this review, including poly(ε-caprolactone) (PCL), polyethylene glycol (PEG), poly (lactic-co-glycolic) acid (PLGA), poly (lactic acid) (PLA), and poly(vinyl alcohol) (PVA), due to their enhanced mechanical properties. Both synthetic and natural polymers are either coated or combined as nanomaterials to form nanocomposite, nano surfaces, nanofibers, and other nanostructures used in BTE [21,22,23,24,25].

### 2.1. Natural Polymers

Commonly, natural polymers are obtained from plants, animals, and microorganisms. These polymers are classified into three main categories: polysaccharides, polypeptides, and polyesters. Therefore, various exclusive characteristics of these polymers (Table 1) for tissue regeneration applications are represented by their superior stability, structural design, tailorable solubility, 3D geometry, excellent biocompatibility, low immunogenicity, antigenicity, cytocompatibility, and frequently specific cell targeting.

**Figure 2:**
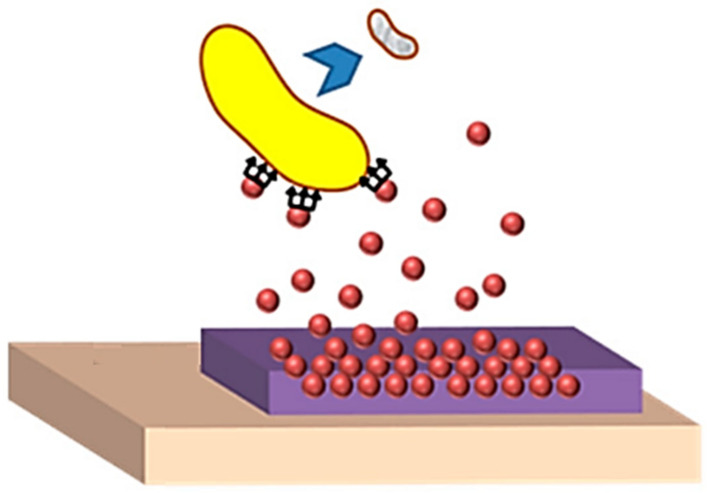
Mechanism of preventing bacterial attachment by using surface coatings [41].

CS is known as one of the most favored materials in BTE due to its biodegradability and non-toxic nature [38]. There are numerous similarities between CS and the native glycosaminoglycans’ structure, which improves the rate of bone regeneration. Thus, Cunha et al. noted that amino and hydroxyl functional groups enable chemical modifications, increasing the potential of CS-based nanocomposites, which have gained significant attention in this domain [39,40]. Such an application can be seen in Figure 2, illustrating the inhibition phenomenon of bacterial attachment.

Additionally, CS is incorporated without difficulty into films, gels, membranes, metallic or non-metallic nanoparticles, as well as composites. This material interacts with negatively charged microbial membranes and, at the same time, exhibits antimicrobial properties against pathogenic microbes. Considering all these aspects, we must also consider the low mechanical strength, unable to support load-bearing applications [41]. Further, Lavanya et al. mentioned that CS can be easily applied as a bone substitute material due to its thermo-responsive capacity and non-toxic abilities. At physiological temperature, injectable CS hydrogels undergo a sol-gel transition, providing improved cellular activity and bone regeneration. Also, these hydrogels facilitate the incorporation of biomolecules, nanoparticles, or polymers, expanding the hydrogel’s properties [42].

Another good example is represented by collagen (Col), an essential component of the bone native extracellular matrix (ECM). Col can be easily obtained from food waste, such as bones, scales, and skins. Further, Col is a natural bioactive polymer with good biocompatibility, facilitating the proliferation and adhesion of osseous cells and reducing antigenicity. In addition, bones’ ECM is composed mainly of Col that nourishes osseous cells and aids the proper transport of nutrients. Even though Col’s biocompatibility enables cell proliferation and adhesion, it also exhibits increased swelling and poor mechanical strength [21]. These limitations are the main reasons to further study the use of Col with various composite biomaterials. Rethinam et al. presented the possibility and applicability of this polymer in the biomedical field by combining it with synthetic polymers or ceramic materials. This research aimed to improve its mechanical properties and bioactivity [43]. Moreover, Marques et Al. presented the applicability of Col-based bioinks for 3D printing in BTE (such as cartilage, bone, and osteochondral regeneration). They have shown that Col is maintained in a liquid state at low temperatures, forming a fibrous structure by increasing the temperature. The solution for this drawback is to combine Col with other materials, thus, improving the rheological properties of bioinks [44].

Gelatin is known as another natural polymer, a processed form of Col, also applied in bone regeneration, including cell attachment of arginine glycine-aspartic acid (RGD) sequences. Even though it is easily biodegradable, this polymer has poor mechanical properties, which are necessary for this type of application [45]. Nevertheless, gelatin can form a thermally reversible network in water, ensuring the formation of a cross-linked network with satisfactory thermal and mechanical stability [46]. Considering these aspects, the mechanical properties of gelatin can be improved by combining it with other polymeric materials or ceramics. To prove this, Luetchford et al. developed scaffolds based on SF and gelatin. After thorough investigation, the obtained scaffolds exhibited improved mechanical strength, and good adhesion of rat mesenchymal stem cells after culturing in an osteogenic differentiation medium [47]. Moreover, Mishra et al. obtained scaffolds based on gelatin and polyvinylpyrrolidone (PVP). The material presented high porosity and interconnected architecture, ensuring a favorable environment to support and aid proliferation and migration using C3H10t1/2 cells [48].

Another preferred natural polymer for BTE applications is hyaluronic acid, which has been intensively used for healing applications due to its good anti-inflammatory capacity. This polymer is well known for its non-immunogenicity, biocompatibility, and biodegradability, promoting cell interaction and proliferation [49,50]. Furthermore, Sionkowska et al. presented the applicability of hyaluronic acid in BTE, drug delivery systems, or coatings [51]. Further, Fang et al. mentioned that hyaluronic acid scaffolds influence several behaviors of stem cells, such as proliferation, adhesion, migration, and differentiation, binding them to specific signaling pathways and cellular receptors. Therefore, researchers focused on developing stem cell- hyaluronic acid systems to improve the efficacy of the composite material [52]. The incorporation of growth factors has been also demonstrated by Park et al. The researchers showed that bone morphogenetic protein-2 (BMP-2) induced the osteogenic differentiation of human dental pulp stem cells and displayed prolonged viability of growth tissue [53].

### 2.2. Synthetic Polymers

Compared with natural polymers, synthetic polymers do not possess the required biological characteristics, which, despite avoiding the generation of an immune response, lead to low capacity, causing detailed cellular phenotypes. Therefore, by incorporating biologically active molecules into synthetic materials, the materials could be designed to fabricate biomimetic scaffolds with well-defined composition, as shown in Table 2. Alternatively, synthetic polymers can effortlessly modify their degradation time, mechanical properties, and molecular weight for BTE applications [23,54].

Furthermore, synthetic polymers do not always generate cell adhesive surfaces and require surface adjustments for suitable growth in 3D architectures. Therefore, the inclusion and encapsulation of cells in coarse conditions reduces cell–biomaterial interactions, limiting its use in bone tissue regeneration [42]. A synthetic polymer appreciated in BTE is PLA, a linear aliphatic thermoplastic material derived from the fermentation of renewable sources rich in starch (e.g., potatoes, corn, or agricultural by-products), making it accessible and available for biomedical applications. Similar to other polymers mentioned above, it has poor mechanical properties, limiting its medical field applicability. In this direction, inorganic particles or other polymers are often incorporated to improve organic polymers’ mechanical properties for bone regeneration [67]. For example, Bal et al. developed scaffolds based on PLA-PEG to properly sustain the incorporation of BMP-2. Still, as the mechanical properties were not sufficient, HA has also been considered to be introduced into the system. The results show the success of its application in BTE [68].

By observing Table 2, it can also be established that PEG is an effective polymer, considering cell adhesion, growth, and proliferation, with good biocompatibility and non-immunogenicity [69,70]. This synthetic polymer has been intensively used in various tissue engineering applications and drug delivery systems. In this regard, Bose et al. mentioned that PEG is preferred in osseous regeneration, as the material can alleviate poor tissue adsorption and rapid metabolism. Further, even at low concentrations, PEG forms a dense, protective, hydrophilic coating, with long flexible chains, leading to a decrease in surface hydrophobicity to the drug molecule [71]. This polymer can be combined with other materials to improve its efficacy in orthopedic applications. Dethe et al. demonstrated the performance of PCL-PEG scaffolds to form fast and reversible physical gelation performance with good bioactivity [72]. Moreover, Bai et al. introduced roxithromycin into a similar system, to prevent inflammatory response at the implanted site. The biological assessments proved good viability and cell growth capacity [73]. On the other hand, Etminanfar et al. fabricated scaffolds containing both polymer PCL-PEG and HA. The obtained material exhibited good biocompatibility and porosity [74].

Another polymer, widely applied in the biomedical field, is represented by PVA, due to its inherent low toxicity, high hydrophilicity, and biocompatibility. Hence, Chen et al. proved that scaffolds based on PVA with β-tricalcium phosphate (β-TCP) provided good thermal processability, while the material presented interconnected channels. Further, in vitro studies demonstrated good biocompatibility, cell proliferation, and adhesion [75,76]. HA is one more ceramic often combined with PVA. For example, Salim et al. obtained composites based on PVA and HA, which significantly enhanced the mechanical and thermal stability, delaying the swelling index. Moreover, the addition of a natural polymer (CS) into the system enhanced the antimicrobial activity and swelling index [77].

Another alternative of synthetic polymer is represented by PCL, a semi-crystalline aliphatic polyester, exhibiting good biodegradability and mechanical properties. Compared with other materials, PCL exhibits great flexibility at a low melting temperature and a slow biodegradation rate for long-term application [78]. Even with these advantages, and it being recommended in tissue regeneration applications, this polymer exhibits poor cell adhesion, proliferation, migration, and low hydrophilicity [79]. To address these issues, PCL can be combined with numerous organic or inorganic materials to surpass the abovementioned limitations. In this regard, Wang et al. obtained a scaffold based on PCL and β-TCP to be further applied for 3D printing. The complex structure was ideal for cell growth promotion, permitting cells to enter through the pores, grow, and progressively cover the pores, improving the osteogenic performance of the scaffold [80]. The same results were also supported by Gatto et al., by developing a scaffold based on PCL that also contained a ceramic material (HA) [81]. Moreover, Cestari et al. demonstrated that by replacing the commercial HA with one derived from seashells, the composite presented enhanced the bioactivity [82].

PMMA is a well-known polymer applied in bone cement, widely used to fixate implants in trauma treatments or orthopedic surgeries [83]. This thermoplastic polymer presents numerous advantages, such as quick polymerization, fast patient recovery, and facile application, but it also presents significant drawbacks. The main disadvantages are the poor mechanical properties and low bioactivity [84,85]. Furthermore, PMMA is a bioinert material and prevents osseointegration and chemical bonding at implanted sites. To surpass these disadvantages, this polymer has been combined with HA, increasing the interfacial shear strength and bioactivity of the developed material [86]. Moreover, Al-Sherify et al. demonstrated that the composite material had a decreased corrosion rate compared with simple polymeric scaffolds [87]. Other researchers focused on other routes, by combining PMMA with other polymers (natural or synthetic). Thus, Atila et al. presented the fabrication of SF/PMMA membranes. The obtained composite showed improvement, considering the mechanical properties and suitable porous architecture. Moreover, biological investigations exhibited good cell attachment and proliferation [88].

## 3. HA-Based on Natural Sources Used in BTE

**Figure 3:**
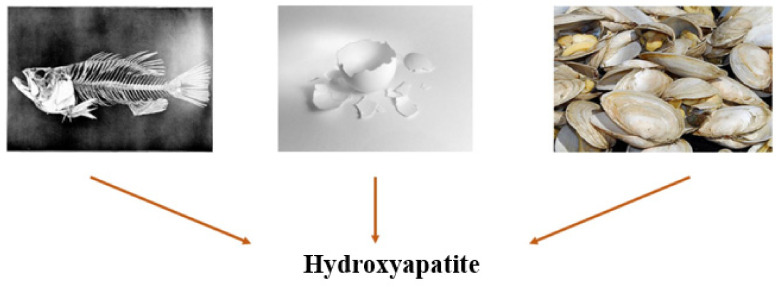
Natural sources of CaPs.

Materials based on CaP attracted the attention of researchers for their effective use in hard tissue engineering. In this direction, HA is a major component for developing coatings, presenting properties such as non-toxicity, osteoconductivity, and increased biocompatibility. Further, HA can be synthesized from chemical solutions containing calcium and phosphate ions. In numerous studies, chemically synthesized HA has been used for bone replacement. Their nanostructures (e.g., nanoparticles, nanotubes) have been obtained through a simple precipitation method. Still, synthetic HA does not show a substantial similarity to native HA, considering its chemical composition, resistance, and bioactivity. In this regard, researchers have been trying to discover the use of natural materials as a substitute source for HA. Several research papers have demonstrated the successful preparation of HA using natural sources, as shown in Figure 3, such as corals, fish scales, eggshells, fish bones, seaweed, animal bones [89,90,91].

Biomineralization is a complex process, widespread in nature, that leads to the formation of a variety of inorganic minerals from living organisms [9]. The minerals produced from biological pathways, with stimulating properties and precise hierarchical structures, are organic–inorganic composites, designed by a self-assembled process in mild conditions (such as temperatures below 100 °C or aqueous solutions) [92]. Numerous studies have shown the potential of converting food waste into extremely valuable bioceramics, using simple and efficient approaches. Different synthesis techniques have been extensively established using animal bones, eggshells, fish bones, oyster shells, or corals. These types of ceramics are presented in Table 3. Further, Rocha et al. demonstrated the conversion of natural aragonite from cuttlefish bone in HA, through the hydrothermal method [93,94]. HA obtained from biological sources generally retains various properties from the precursor materials, such as chemical composition and pore structure. In this direction, it possesses a rich calcium concentration and beneficial trace elements, such as Mg and Na, for bone growth [95].

In addition, bio-waste, including eggshells, animal bones, and marine shells, have shown great potential in this path. The use of bio-wastes is economical, helps sustainability, and adds value [114,115]. In recent years, the possibility of preparing HA from fish bones has been demonstrated by simple calcination. The HA obtained through this process has a structure and morphology very similar to human bone. Furthermore, by subjecting it to a high-temperature treatment, all pathogens and organic components from the source are removed. Furthermore, HA extracted from natural sources generally contains traces of ions, such as Mg^2+^, Na^+^, Zn^2+^, and K^+^. These ions enhance the usefulness of natural HA, promoting the regeneration processes and bone formation [116].

**Figure 4:**
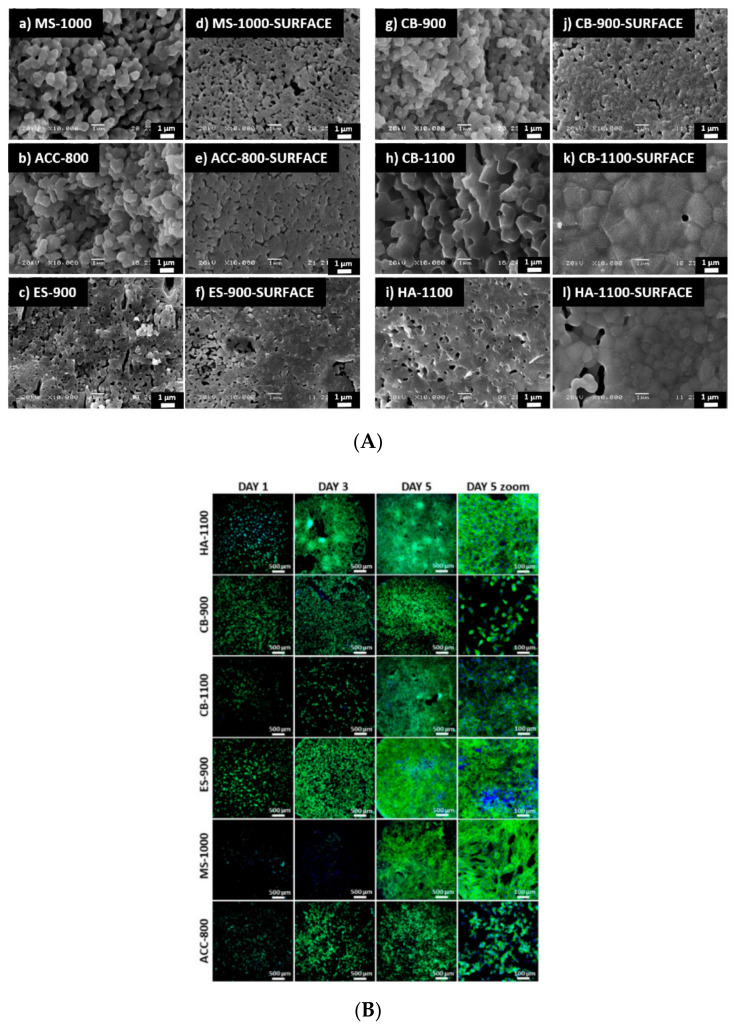
(**A**) SEM images of fracture and external surfaces, (**B**) Confocal images of adhered cells on the sintered pellets of obtained materials. Notation corresponds to the provenience of the natural source and sintering temperature: eggshells (ES), cuttlebones (CB), mussel shells (MS), and amorphous calcium carbonate (ACC) [117].

Cestari et al. obtained nano-HA from different sources (eggshells, cuttlefish bone, and mussel shells). HA was synthesized via wet mechano-synthesis and drying in an oven. The powders were further mixed with phosphoric acid (~85% H_3_PO_4_), or ammonium phosphate dibasic ((NH_4_)_2_HPO_4_), to achieve a Ca/P ratio of 1.67. The results of their study are presented in Figure 4. After a thorough investigation, researchers concluded that HA obtained from food wastes exhibited good cell adhesion and proliferation after seeding through confocal microscopy. SEM micrographs of the obtained materials also show that most pores have dimensions ~1 µm; thus, it can be considered that they are not perceived as holes by the cells, dimensions of which are up to100 µm. Nevertheless, the micrometric pores are beneficial for cell–bioceramic interaction [117].

All prepared samples proved good cellular adhesion with no cytotoxic effect. Moreover, HA derived from eggshells and sintered at 900 °C encouraged the greatest cell adhesion pattern, comparable with pure HA, without forming clusters. At the same time, those from mussel shells and cuttlebones also have the capacity to support cell adhesion, while amorphous calcium carbonate-based HA displayed lower cell-to-cell interconnection and round-shaped cells.

### 3.1. Recent Strategies for Obtaining HA from Natural Sources

Recently, new, sustainable methods for obtaining HA with particular properties have been extensively studied. Although numerous synthesis methods were developed, HA’s preparation with specific properties remains a challenge, due to the risk of appearing toxic intermediates during synthesis [118]. Hence, several chemical methods have been chosen to obtain HA in this field: hydrothermal techniques, precipitation, wet chemical precipitation, microwave processing, and hydrolysis. HA obtained from marine species has great economic influence over commercial HA due to the low-cost synthesis technique. Moreover, natural sources possess the advantage of taking over certain natural material properties. Nowadays, numerous compounds are isolated from marine organisms and are applied in bioactive ingredients on medical devices [119,120,121,122].

Different synthetic approaches have been applied to prepare HA-based composites, varying their properties according to the application’s necessities. Solid-state synthesis is a well-known technique, used to obtain inorganic oxide materials, because it allows excellent control of the chemical stoichiometry. However, challenges arise when the formation of the phase composition of the anticipated product is not compatible with the thermal treatment and high temperatures to obtain a complete solid-state reaction [123]. Depending on ionic strength, precipitation conditions, pH, and temperature, HA is obtained with a Ca/P molar ratio similar to natural bone, making it an ideal candidate for clinical applications [124]. However, these methods can be very complex and time-consuming. HA synthesized from animal bones presents the advantage of retaining certain raw material properties, such as composition and chemical structure [125,126]. The ball milling method was also preferred to mill the fish bones before performing the calcination process. The resulting powder was produced within a well-established time frame, which can influence the purity of the ceramics obtained [127].

In this direction, Yanhong et al. suggested an ecological and green method to extract HA from fish scales with a deep eutectic solvent (e.g., choline chloride/1,4-butanediol). The ideal extraction conditions were as follows: the extraction temperature of 65 °C, in 2 h, and a specific solid:liquid ratio of 1:15 (g:g). All these conditions lead to a HA extraction of 40.58%. The results showed that the extracted HA presented a heterogeneous morphology, a mixture of phosphates of phosphoric acid and carbonic acid, with excellent thermal stability and high purity. The obtained HA reported a good adsorption capacity for metal ions, such as copper, lead, zinc, and silver [128]. Wu et al. prepared three synthesis methods from three different fruits and eggshells. After performing the hydrothermal reactions at 150 °C for 24 h, aggregate particles with a rod-like or needle-like nanostructure were obtained. Needle-like nanostructures were transformed into rods as the hydrothermal reaction time increased by 72 h.

Moreover, Wu et al. concluded that HA obtained with pomelo peel extract showed the best structure, having the most similar crystalline structure of HA, present in natural bone [129]. Further, Karunakaran et al. reported the influence of PVP. This polymer is used as an organic modifier for HA synthesis. Seashells have been used for HA synthesis as a source of calcium. The microwave-assisted synthesis method was applied to obtain various sizes and complex mesoporous morphologies that can be subsequently applied in implantation. Also, the presence of PVP improved the microwave-stimulated approach, which effectively regulated pore volume, surface area, size, and dimensions by regulating the formation of HA crystals [130].

### 3.2. HA Obtained from Eggshells

This natural method of obtaining HA nanorods is not only an optimal and energy-saving process, but it also excludes high-energy calcination and uses exogenous surfactants to assist the synthesis of HA from shells or oysters as a source of calcium. This obtainment method improved CO_3_^2-^ ions’ loading capacities and enhanced the poor conversion performance of calcite into HA, to some extent [131,132]. The use of shells proved advantageous through the impurities derived from the organic matrix in CaCO_3_. The residual organic matrix of CaCO_3_, derived from mollusk shells, offers biocompatibility and bioactivity to the composite, promoting bone regeneration [133,134]. The quantity of HA improved significantly, and the diffraction peaks’ intensity increased through the ball grinding process. A longer grinding time leads to the improvement of these two properties. Studies have shown that stoichiometric HA powder can be effortlessly obtained through ball milling without further heat treatment. In this regard, Lala et al. showed that 10 h grinding is not enough to obtain pure HA without sintering aids [135].

Eggshells are also a natural mineral source (e.g., calcium carbonate) that can be extracted and used to obtain HA. From these resources, it offers bioactivity and biocompatibility that allow osteoblast cells the capacity to adhere and proliferate in different periods [136,137,138]. Francis et al. showed the applicability of eggshells. They are generally subjected to grinding to facilitate membrane removal, either by heat treatment or by bleaching [139]. Wu et al. described the development of HA powder using dicalcium phosphate dihydrate (DCPD) and eggshell powders through ball milling and following heat treatment. HA phase formation can be started by sintering the sample at 1000 °C for 1 h, while the pure HA phase is obtained by sintering the ground sample after 10 h [140]. Also, Ho et al. proposed eggshells as a possible technology for recycling waste management of materials. Eggshell is also a possible material in CaP ceramics synthesis for biomedical applications. Some trace elements do not modify the elementary crystallographic properties of HA but may improve the general biological performance of the material used for implants [141]. Alhussary et al. applied nano-HA from seashells and eggshells to the damaged mandibular bone of rabbits and reasoned that HA obtained from eggshells has an improved osteoinductivity and porosity than the one obtained from seashells [142]. Furthermore, Waheed recommended that eggshell scaffolds have superior compressive strength, increased mineralization, and improved osteogenic differentiation potential compared to commercial HA [143].

### 3.3. HA Obtained from Seashells

Nanometer-sized ceramics of marine origin provide an abundant source of new materials in BTE but are also a source of stimulation for developing novel biomimetic composites. Natural corals are composed of an organic matrix that presents calcified nodes. The shells consist mostly of calcium carbonate, which forms multilayered microstructures and small organic components (1–5 wt.%), located mainly in the inter-crystalline boundaries. Despite its composition and special composite microstructure, shells show an improvement in strength by three orders of magnitude over synthetic calcium carbonate. [144].

The oyster shells represent another natural source, composed of pure aragonite, which is crystallized in an organic matrix. Vecchio et al. performed a classic hydrothermal conversion process from sea urchin spines into TCP. The obtained products exhibited good bioactivity and osteoconductivity [145]. Researchers demonstrated that rod-shaped HA crystals are favored in the attachment of proteins, especially in the binding of vitamin D, applied in BTE, due to its strong adhesion to osteoclasts and similarity with the native HA structure. Moreover, HA synthesized from microwave-assisted treatments is different from conventional methods. Heat is initiated uniformly into the material for this production method, with a higher heat transfer rate and spatial temperature distribution. Once the sample nears the heating source, the size, shape, and chemical structure develop high sensitivity to heating [146].

**Figure 5:**
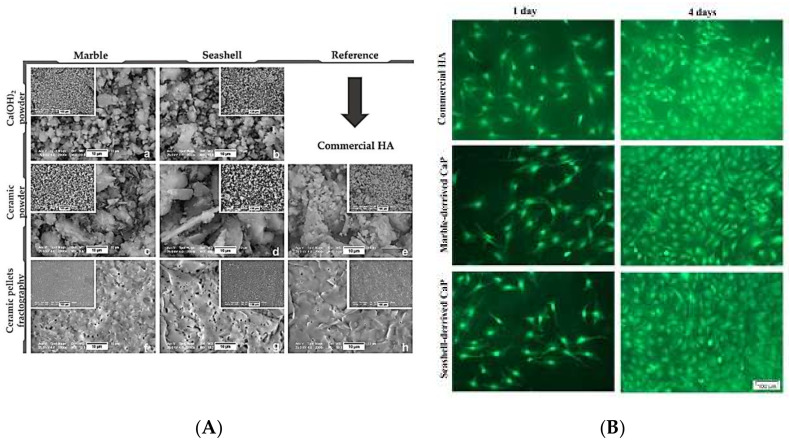
(**A**) SEM images of reference, seashell, and marble materials (on the magnification of 10 and 100 µm); (**B**) Fluorescence microscopy images of the MC3T3-E1 cells [147].

Mitran et al. studied the bioactivity of CaP derived from *Mytilus galloprovincialis* seashells and dolomitic marble. The obtained materials were investigated on MC3T3-E1 pre-osteoblasts, regarding morphology, viability, cell adhesion, proliferation, and differentiation, to investigate their potential for BTE (as shown in Figure 5). Results exhibited suitable cell adhesion and high viability, with no distinguishable differences in the morphological features. Moreover, the pre-osteoblast proliferation on naturally derived CaPs showed a substantial increase compared with commercial HA. Nevertheless, seashell-derived CaP displayed a higher efficiency in promoting pre-osteoblast differentiation and further exhibited its potential in BTE [147].

### 3.4. HA Obtained from Fish Bone

While synthetic materials have been known for their use in medical applications, natural materials are nowadays acknowledged as a potential candidate for new biomedical applications. Given that fish bones are abundant materials, rich in CaP, their exploitation has certainly been beneficial in reducing disposal costs and reducing the risk of environmental pollution, in addition to their biomedical benefits [12,148]. Numerous species, such as codfish (*Gadus morhua*), barramundi (*Lates calcarifer*), carp (*Cyprinus carpio*), cuttlefish (*Sepia Officinalis*), golden (*Sparus aurata*), conger (*Conger conger*), perch (*Dicentrarchus labrax*), flatfish (*Heterosomota pleuronectiformes*), sardines (*Sardina pilchardus*), anchovies (*Engraulis encrasicolus*), sharks, swordfish (*Xiphias gladius*), tuna (*Thunnus albacares*), have been applied as raw materials to produce HA and β-TCP [12]. For example, Ozawa and Suzuki prepared HA from Japanese sea bream by calcination at 1300 °C [149]. Calcination time, calcination temperature, extraction method, and bone nature are aspects that influence the final properties of CaPs, such as morphology, Ca:P ratio, size distribution, phase purity, and specific surface area [150].

Moreover, CaPs derived from fishbone have been shown to accomplish functions similar to commercial ones. Fish bones would make a significant input to developing biomaterials for BTE. They possess great potential in developing several products that could be used in many applications, including tissue engineering, environmental remediation, and drug delivery [12,151]. Buraiki et al. studied the synthesis of pure HA from fish scales. The results confirm the synthesis of HA powder, which could be further used for bone regeneration [152]. Another good example is represented by the study performed by Piccirillo et al. The research group examined the annealing of codfish bones at different temperatures of 900, 950, 1000, 1100, and 1200 °C. The final materials obtained were composed of β-TCP and HA. Hence, the temperature is another property that greatly influences the process of obtaining ceramics. The β-TCP content increased by increasing annealing temperature [153]. Goto and Sasaki also examined the influence of the temperature (400–1000 °C) on bone calcination. It was demonstrated that the diameters of the crystallite samples increased, whereas their specific surface area decreased as the calcination temperature increased [154].

HA powder obtained using heat treatment on fish scales (*Catla catla*) retained, in its composition, natural trace elements, such as magnesium (Mg) and strontium (Sr), with a concentration of ~3.5 times more Mg and ~10 times more Sr, related to chemically synthesized HA powder [112]. Fish-derived HA crystallite of nanometric dimensions was investigated compared to chemically synthesized HA. In this regard, the average crystallite size and HA crystallinity derived from fish increased with the calcination temperature. From the point of view of biological analyses, in vitro cellular materials’ interactions with osteoblast cells showed no toxicity [112,155]. The porosity of HA from marine resources is higher compared to ceramics obtained by chemical methods. The increased porosity is considered a great advantage of its application in biomedicine. The values of synthesized HA hardness are in the range of human cortical bone [156].

**Figure 6:**
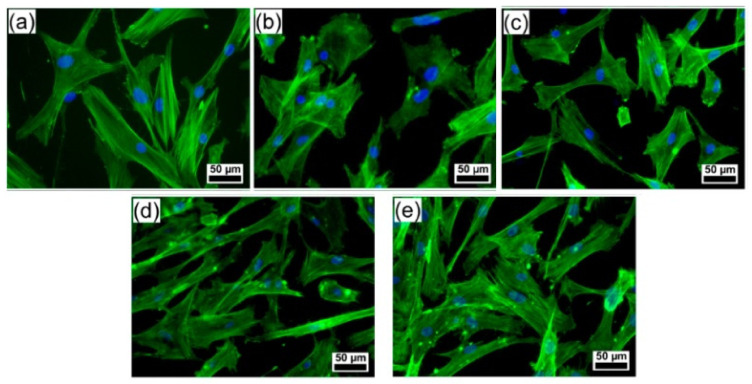
Fluorescence microscopy images of human gingival fibroblast cells morphology on the surface of (**a**) control (polystyrene surface (**b**) simple Ti, (**c**) commercial HA, (**d**) sea bream-BCP, and (**e**) salmon-BCP coatings [157].

Popescu-Pelin et al. presented the applicability of fish bone-derived BCP for obtaining thin films via pulsed laser deposition (PLD). The targets were prepared from sea bream and salmon fish bones for this experiment. In the obtained coating, a significant fraction of β-TCP was observed in their composition besides HA, which contributes to improved biocompatibility and solubility for BTE. The deposited structures were biocompatible and presented no signs of cytotoxicity in human gingival fibroblast cells, demonstrating their increased osseointegration (Figure 6). Further, the composite inhibited microbial adhesion and biofilm development. Moreover, protection against bacterial colonization of *Escherichia coli* was established for at least 72 h, due to the release of their natural elements (i.e., Mg, Na, S, Si) from fish bones [157].

Moreover, to support the use of fish bones, Bas et al. investigated the in vitro biocompatibility of Salmon-derived BCP, by performing the characterization on osteosarcoma (Saos-2) cells. All results have shown that the obtained material had no cytotoxicity and promoted their applicability [158]. In vitro biocompatibility was also examined in the behavior of osteosarcoma (Saos-2) cells, indicating that the CaP bioceramics had no cytotoxicity effect. Salmon-derived biphasic calcium phosphates (BCP) have the potential to contribute to the development of bone-substituted materials.

## 4. Composite Materials: Polymers and HA Based from Natural Sources

It is well known that human bone consists mainly of HA crystals and collagen fibers. Synthetic CaPs have attracted considerable attention due to their functional and structural similarity to natural HA. Their superior biocompatibility and excellent osteoconductivity have increased their popularity in recent decades, but they did not exhibit osteoinductivity. Some approaches implemented to bestow osteoinductivity include ion substitution, combining them with various polymers to obtain a composite, or adding bioactive substances [159]. BTE applications are designed to repair defects by introducing scaffolds into the human body, which are consequently replaced by novel tissue. For these applications, nanocomposite biomaterials are preferred to incorporate a matrix structure with good bioactivity, easily resorbable [160,161].

In this regard, hydrogels enable the mineralization throughout bone regeneration processes with bioactive inorganic materials. Nanohydroxyapatite (nHA), CaPs, and bioglasses are among the most extensively used bioactive inorganic materials to be doped into hydrogels [162]. An example of such applications is presented by Deb et al., in which the team developed scaffolds composed of HA from fishbone into a PMMA matrix. After thorough investigation, the obtained scaffolds proved to meet the physiological requirements but also necessary in vitro bioactivity [163]. Another composite is presented by Pon-On et al., by obtaining PLA/CS composite scaffolds with HA from fish scales, with good bioactivity and cell adhesion [164].

### 4.1. Properties of Composite Materials for BTE

**Figure 7:**
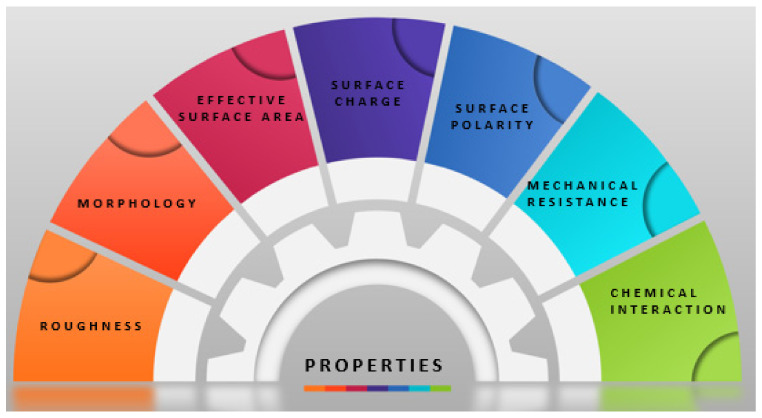
The connection between the properties of the material applied as a surface coating [167].

The mechanical properties of materials (Figure 7) depend on their specific surface, total pore volume, pore size, pore distribution, and porosity. A ceramic with high porosity, inhomogeneous pore size distribution, large pore size, superior total pore volume, and large specific surface area will exhibit low strength. Pores decrease the strength of the ceramic, reducing the cross-sectional area when a load is applied. Thus, reducing the porosity, specific surface area, pore size, and total pore volume will improve ceramic strength [165,166].

As observed in Figure 7, the implant’s surface influences the bacteria’s ability to adhere to the substrate. The application of surface coatings is the optimal technique to prevent bacterial attachment and increase the bioactivity of the implant. Moreover, numerous studies have also demonstrated that the efficiency of the applied coatings is influenced by the materials’ properties used in their development. One of these properties is surface morphology. The roughness of the surface particularly affects the behavior of bacterial adhesion. Even though a polished surface presents the capacity to reduce bacterial adhesion, roughened surfaces offer encouraging biological properties with good biomechanical stability. Surface hydrophilicity also has a major impact on bacterial adhesion. As the roughness is reduced from sub-micron to nano-scale dimensions, the surface properties are progressively modified from hydrophobic to hydrophilic, becoming unfavorable to the adhesion of *S. aureus*, which is hydrophobic. Moreover, the bacterial adhesion, respectively, the bond between the bacteria and surface deteriorates [168,169].

A promising method to prevent biofilm formation from an early stage is the use of antimicrobial surfaces. Surfaces doped with antimicrobial elements (such as copper, silver, and zinc) are developed to surpass antibiotic delivery deficiencies [170,171,172]. The latest tendency in biomaterial science is the combination of polymeric and ceramic materials. The main disadvantage of ceramics is their fragility. Flexibility can be improved by incorporating inorganic compounds into polymeric matrices. Current research involves the development of different composite materials by incorporating HA into the system. It is well known that HA has great compatibility with osteosarcoma cells. However, it is still necessary to design polymer matrices that also show improved biocompatibility in contact with human cells [173]. To induce the preferred mechanical strength, HA synthesis often requires a high temperature for the sintering process, so ceramic materials, developed by processing at low temperatures, are very fragile and unsuitable for tissue engineering applications. The use of ceramics (e.g., HA) in scaffold development has two major disadvantages: the deficiency of degradability in biological systems and limited processing techniques for manufacturing scaffolds (Table 4).

In general, for biomedical applications, the benefits of ceramic materials have many more advantages than limitations [179]. The ideal property of implants is to exhibit antimicrobial activity, to reduce the treatment period, showing a local antibacterial effect and improving its effectiveness, thus, reducing the need for systemic therapies and their side effects [180].

### 4.2. Applications of HA-Based Composites in BTE

Biomaterials applied in the biomedical field have many chemical and physical properties. Biomedical applications in the surgical and medical domain require a thorough understanding of their mechanical properties. An exhaustive investigation of the mechano-tribological properties of implanted materials is necessary to determine the reliability in the long term [181]. With the progress of research in recent years, researchers focused on providing solutions to combat biofilm and, at the same time, to maintain osseointegration [182]. There are various methods to address this issue, especially the fabrication of 3D scaffolds, micro or nanoparticles, and the in situ formation of hydrogels that are applicated to design substitutes of the natural tissue, encouraging regeneration [175]. Nevertheless, it is well known that composite materials containing ceramic and polymer constituents have combined properties, such as bioactivity, biodegradability, and flexibility from polymers, but also mechanical strength and toughness provided by the ceramic phase. Except for their biocompatibility, materials applied in BTE also need to support cell viability, proliferation, and differentiation. Moreover, the porous structure ensures satisfactory space to promote bone ingrowth. The macropores facilitate the osteoid formation and mineralization, increasing the osteoblasts and osteoprogenitors migration into the scaffold, while the interconnected microporosity enhances vascularization and nutrient distribution throughout bone reconstruction [183].

Until now, little evidence has been presented regarding the positive properties of HA from natural sources on human health, because of the absence of comprehensive investigation and experimental research. Few researchers have effectively obtained HA-based biomaterials from various food wastes, such as scales, bovine and fish bones, eggshells, and seashells, and performed the corresponding characterization analyses [184]. Recent advances in biomaterial synthesis, especially those applied in the development of medical applications, to prevent bacterial infections and improve osteogenesis, have reawakened natural HA’s research interest. [185,186]. Mahmoud reported alginate/nano-HA composites’ preparation, using fishbone as a natural source for HA extraction. The 3D porous scaffold was manufactured to improve biodegradability and osteoconductivity. The animals studied to assess biological effects survived without extensive or local complications, which means that the implanted materials did not generate any histopathological limitations or non-compatibility with bone tissue [187]. Moreover, Yadong et al. found that combining HA and Col improves the differentiation of osteoblasts, due to their excellent bioactivity, biocompatibility and osteoconductivity. Therefore, the Col/HA structures showed a microstructure and composition similar to natural osseous tissues, attracting great potential candidates [188].

In another study, the twin-screw blending solvent casting process obtained biofilms. The polymer chosen for this application was PLA, a biodegradable polymer, while HA is obtained from fish bones. The obtained PLA/HA biofilms were characterized by several characterization methods, such as XRD, FESEM, DSC/TGA, FTIR, and contact angle. The results obtained were very encouraging, offering the possibility to fixate medical devices to bone tissue. Moreover, XRD confirms that the presence of HA generates a higher crystallization of the films [189].

Further, novel CS/CaP-based composites have been developed to regenerate bone tissue ad were studied by Kara et al. Initially, fish residues are physically fragmented and effectively decellularized into microparticles, and further incorporated into the matrix of CS. The results presented extremely porous composites, in which the ceramic microparticles were incorporated as fibrillar structures, with diverse morphologies into the CS surface. After 14 days, it could be observed that the addition of natural ceramics did not modify the composite’s mechanical properties. Moreover, their morphology changed the polymeric matrix structure and surface roughness, enhancing cell attachment [190]. In another study, Humayun et al. developed an antimicrobial coating, based on CS, combined with halloysite nanotubes, as an ideal solution to avoid biofilm formation, because of the innate biocompatibility of halloysite and ability to offer controlled drug release. As a bioactive principle, gentamicin is used to enhance its further biocompatibility [191].

Another innovative method is presented by Kang et al. The researchers obtained calcium-binding polymer-coated poly(lactide-co-glycolide) microparticles, which incorporate a quorum sensing (QS) reagent (Furanone C-30), and attached them to the HA surface to prevent biofilm formation. After thorough analysis, they showed a good adhesion of the microparticles on the surface of the material and demonstrated that the microparticles containing furanone C-30 can inhibit biofilm formation, generated by *S. mutans,* up to 18 h [192]. HA-coated titanium implants can induce the complete osseointegration of bone tissue [193]. These coatings on implant surfaces can be obtained through numerous techniques, such as electrophoretic deposition, plasma spraying, sol-gel deposition, sputtering deposition, pulsed laser deposition, spin-coating, ion beam assisted deposition, and many other techniques [183,193].

Another innovative application is represented by 3D printing technology, which recently engrossed extensive attention in BTE. In this direction, HA particles and multipotent mesenchymal stromal cells are directly added to the Col solution and then applied to different molding methods. Li et al. mentioned the success of combining mineral ions with Col, followed by mineral formation through an in-situ deposition. This technique can be applied to control the regular arrangement of fibers in scaffolds [194]. Further, Lowe also studied the possibility of forming a new composite based on salmon bone HA, functionalized with fucoidan and CS as 3D scaffolds. Nano-HA was isolated from the salmon fishbone through alkaline treatment and further uniformly dispersed in the polymer matrix. The composite pore size was 23–354 μm, suitable for accompanying growth factors and nutrients [195].

All these studies presented the possibility to use composite materials in tissue engineering applications to improve osseous regeneration, without exhibiting any harmful effects.

## 5. Future Perspectives and Challenges in BTE

BTE has progressed extraordinarily considering materials synthesis, application techniques, and cell engineering, due to the increased demand in the medical field. The main goal in this domain was to improve the treatment of bone defects without applying autologous bone grafts. Until this moment, the ideal material to simulate natural bone regeneration has not yet been obtained. The development of composite scaffolds based on natural sources appeared to be the perfect solution to be further applied and improve the biological activity, without the appearance of side effects. In this regard, the fabrication of artificial bone grafts, composed of bioceramics and biopolymers, represents the main solution in bone tissue regeneration [196,197,198]. Even though researchers came up with solutions of synthesis, the obtained biomaterials still showed poor mechanical properties compared with natural tissues. Moreover, synthetic tissues exhibited a lower integration and degradation rate in clinical applications. Numerous domains, including biology, materials science, and biophysics, combined their work in BTE, to interwind polymer synthesis and cell treatment to develop the ideal methods to produce novel materials [199,200]. The interaction between scaffolds and cells is a crucial aspect in designing scaffolds for tissue regeneration applications [201].

Considering bioceramics, HA materials can be improved through doping with several nonmetallic or metallic dopants to enhance their properties for further use. For example, Mg prevents HA crystallization, necessary for bone formation, and decreases bone fragility. This dopant can replace ~1% Ca^+2^ ions, presenting similar properties to bone tissue. Further, Zn disturbs the stability of the solution, inhibiting bacterial colonization, but also regulates inflammatory mediators. Another dopant, Sr, inhibits bone resorption, promotes osteogenesis, activates signaling pathways, and regulates cell behavior [202,203]. The doped ceramics can be further applied in combination with polymers to avoid the problems concerning biocompatibility and biodegradability; thus, the increase in biological activity. To explain the biological effects, scaffolds used in BTE are investigating angiogenesis, immunoregulation, biomineralization, and osteogenesis as the osseous tissue regeneration process. Although these materials were thoroughly investigated for biomedical applications, there are still many concerns for in vivo suitability and cytocompatibility. Nevertheless, the main challenge is to optimize biomaterials to further use them in manufacturing processes [204].

On the other hand, biofabrication techniques led to the formation of improved scaffolds, with better properties, but still insufficient to recreate the complex structural organization of organs or tissues. This challenge is crucial for the clinical translation of BTE applications and approval in the medical field. Most concerns have been focused, particularly, on the process and applied technology. Additive manufacturing methods (e.g., stereolithography, fused deposition modeling, selective laser sintering, 3D bioprinting) are the most applied techniques in bone regeneration. The main advantage, when one of these methods is used, is that scaffolds are dimensioned directly, reproducing the required 3D space. Moreover, this enables biologists to process precisely inorganic and organic materials [204,205].

## 6. Conclusions

In this review, the latest materials applied in bone tissue regeneration have been investigated to select the suitable candidate, designed as scaffolds with ideal properties. Firstly, natural, synthetic polymers and their applicability have been discussed, considering their advantages and drawbacks. Moreover, the possibility to obtain HA from natural sources was also studied, due to its similar structure with the native HA. The combination with polymeric materials (e.g., Col, CS, PLA, gelatin) can lead to an increase in the scaffold’s bioactivity and applicability in BTE.

Taking into consideration the possibility to combine polymers and ceramics, to tailor the properties of the composites, the characteristics of the ideal material have been researched to be subsequently applied in the medical field. In this direction, the possibility of using composite materials containing HA derived from natural sources has gained great attention for their application as scaffolds, surface coatings, or bio-inks, for 3D bioprinting. All the presented studies offered a general background in selecting the optimum material to be further applied in BTE, while the biologic activity has not yet been fully discovered.

This study’s final goal is to identify the natural source for HA and the suitable polymer, which can be combined with the selected ceramic. Currently, the main challenge in hard tissue engineering is optimizing the material synthesis method by augmenting the biologic activity and their physicochemical properties, without causing any negative effect.

## Figures and Tables

**Table 1:** General characteristics of natural polymers and their applications in BTE.

Polymer	Properties		Applications	References
	Advantages	Disadvantages		
Collagen	Great biocompatibility, biodegradability, cytocompatibility, non-toxicity	Poor mechanical strength	Scaffolds, drug delivery systems, 3D printing	[26]
Alginate	Biodegradability, biocompatibility, bioresorbability, non-toxicity, presenting synergic effects with bioactive components	Poor mechanical strength and bioactivity	Bone tissue applications	[27,28]
Chitosan	Superior biocompatibility; biodegradability, anti-inflammatory	Poor stability, mechanical strength	Hydrogels, scaffolds, microspheres	[29]
Hyaluronic Acid	Great biocompatibility, biodegradability, cell adhesion, proliferation, and differentiation	Poor mechanical properties, high degradation rate	Scaffolds, hydrogel	[30,31,32]
Bacterial cellulose	Good water absorption, mechanical strength and structural properties, good cell adhesion and biocompatibility, continuous structural support	Low biodegradability in the human body and biological activity	3D scaffolds, bone tissue replacements	[33,34]
Silk fibroin	Increased flexibility, biocompatibility, with good mechanical strength	Reduced biodegradation rate	Scaffolds	[35]
Gelatin	Great biocompatibility, biodegradability, non-toxicity, improved cell adhesion, and proliferation	Poor mechanical properties, high biodegradation rate	Scaffolds for hard tissue engineering	[26,36,37]

**Table 2:** General characteristics of synthetic polymers and their applications in BTE.

Polymer	Properties		Applications	References
	Advantages	Disadvantages		
Polylactic acid (PLA)	Superior tensile strength, elongation, and modulus, biodegradability, and minimal inflammatory response	Low toughness, mechanical support, insufficient biocompatibility	Load bearing applications, orthopedic repair, suture anchors, scaffolds	[27,55,56]
Poly(ε-caprolactone) (PCL)	Good biodegradability, biocompatibility, low Young’s modulus, tailorable physical properties, reduced degradation rate	Poor cell adhesion, hydrophobic nature	Scaffolds, BTE, 3D bioprinting	[55,57,58]
Poly(glycolic acid) (PGA)	High crystallinity; great mechanical strength, good cell adhesion, proliferation, and differentiation	Hydrophobic nature	Scaffolds, BTE	[58,59]
Poly(vinyl alcohol) (PVA)	Biocompatibility, biodegradability, good compressive mechanical and elastic strength	Low bioactivity, decreased cell attachment	Scaffolds, drug delivery systems	[60,61,62]
Poly(ethylene glycol) (PEG)	Biocompatibility, hydrophilicity, able to improve degradation, non-toxicity, and non-immunogenicity combined with different polymers, enhanced enzymatic stability	Limited tailorable mechanical property and rheological behavior, reduced bioactivity	Scaffolds, BTE, 3D bioprinting, orthopedic implant	[58,63,64]
Poly(lactic-*co*-glycolic acid) (PLGA)	Excellent biocompatibility, processability, good mechanical strength, adjustable degradation rate, and minimal inflammatory response	Possible inflammatory response, low bioactivity	Scaffolds, orthopedic implants, drug delivery systems	[65,66]
Poly(methyl methacrylate) (PMMA)	Processability, durability	Non-degradability	Scaffolds	[66]

**Table 3:** Examples of CaPs obtained from natural sources.

Natural Source	Crystalline Phase	Morphology	Application	References
Fishbone	Hydroxyapatite	Laminar and irregular structure, 149–325 nm	Surface coating; nutrition	[96,97,98,99]
	Biphasic calcium phosphate	30–100 nm, as nanorods	Scaffolds	[100]
Eggshells	Biphasic calcium phosphate	Spherical structure	Orthopedic and dental applications	[101]
	α-Tricalcium phosphate	Compact and agglomerated structure	Scaffolds; dental reconstruction	[102,103]
	ꞵ-Tricalcium phosphate	Round shape, with dimensions between 150 nm–2 µm	Scaffold in dental and orthopedic reconstruction	[104]
	Hydroxyapatite	Irregularly shaped, with sizes between 10–18 µm	Reinforcing filler; biomedical devices	[105,106]
	Hydroxyapatite	Flower-like, with the aspect of hexagonal rods and dimensions between 200–300 nm	Biomedical applications	[107]
Seashells	Hydroxyapatite	Nano-rods, with sizes between 20–90 nm	BTE; drug delivery; dentalApplications; coating	[108,109]
Fish scales	Hydroxyapatite	Dimensions between 20–60 nm, in the form of agglomerations or nano-rods	Coating; dental applications; bone graft; filler	[110,111,112,113]

**Table 4:** Required properties of scaffolds in BTE applications.

Property	Required Characteristics	References
Biodegradability	The material should possess a prearranged biodegradability to improve the composition of different tissue. In this manner, the biodegradable matrices offer temporary scaffolds within defects into the bone tissues to improve their regeneration.	[174]
Biocompatibility	The composite material must perform with a suitable host response in the regeneration of bone tissue. This ability must be in synchronization with osseous tissue without producing damaging changes.	[175]
Mechanical Properties	Surface roughness enhances cell attachment, differentiation, and maturation. Moreover, scaffolds’ mechanical stability supports their adhesion to the neighboring tissue. These properties enhance the adsorption of adhesive proteins (e.g., fibrin), leading to an improved osteogenic cell attachment, proliferation, and differentiation into osteoblasts, to further bone production integrated within the scaffold.	[55,176]
Porosity	Needs be tuned, as the initial porosity must be low or else the scaffold resorbs very fast, incapacitating the mechanical support to further affect novel tissue growth. On the other hand, materials with a low degradation rate can possess high porosity, optimizing the degradation of the scaffold.	[177]
Bioactivity	This characteristic is essential to improve ECM development through the stimulation of cellular behavior andcan contribute to the cells the molecular signals.	[174,176]
Processability	The composite material should be easily processed to design various formulations and configurations such as nanometric, 3D scaffolds, micro-metric particles, and/or injectable formulations.	[175]
Immune response and toxicity	The obtained materials must be non-cytotoxic and allow cell attachment to function properly, proliferate and differentiate. Moreover, they must possess non-inflammatory properties and induce a minimal immune response.	[178]
Controlled Delivery	To deliver biomolecules in BTE applications, it is mandatory to develop scaffolds as a drug delivery system. Additionally, the biological activities of these biomolecules and interaction among surrounding cells in the bone-healing process are the foundation for the fabrication of BTE scaffolds.	[176]

## Data Availability

Not applicable.
